# Specific localisation of ions in bacterial membranes unravels physical mechanism of effective bacteria killing by sanitiser

**DOI:** 10.1038/s41598-020-69064-1

**Published:** 2020-07-23

**Authors:** Judith Thoma, Wasim Abuillan, Ippei Furikado, Taichi Habe, Akihisa Yamamoto, Simone Gierlich, Stefan Kaufmann, Klaus Brandenburg, Thomas Gutsmann, Oleg Konovalov, Shigeto Inoue, Motomu Tanaka

**Affiliations:** 1 Physical Chemistry of Biosystems, Institute of Physical Chemistry, University of Heidelberg, 69120 Heidelberg, Germany; 2 Analytical Science Research Laboratories, Kao Corporation, 1334 Minato, Wakayama, Wakayama Prefecture 640-8580 Japan; 3 Center for Integrative Medicine and Physics, Institute for Advanced Study, Kyoto University, Kyoto, 606-8501 Japan; 4 Research Center Borstel, Leibniz Lung Center, 23845 Borstel, Germany; 5 European Synchrotron Radiation Facility (ESRF), 38043 Grenoble, France; 6 Present Address: Brandenburg Antiinfektiva GmbH, 23845 Borstel, Germany

**Keywords:** Chemical physics, Biophysical chemistry

## Abstract

Antimicrobial resistance is a major threat to public health. Although many commercial sanitisers contain a combination of cationic surfactants and aromatic alcohols, the physical mechanisms where these two substances bind to or how they disturb bacterial membranes are still largely unknown. In this study, we designed a well-defined model of Gram-negative bacteria surfaces based on the monolayer of lipopolysaccharides with uniform saccharide head groups. Since commonly used X-ray reflectivity is sensitive to changes in the thickness, roughness and electron density but is not sensitive to elements, we employed grazing incidence X-ray fluorescence. In the absence of Ca^2+^, cationic surfactants can penetrate into the membrane core with no extra support by disturbing the layer of K^+^ coupled to negatively charged saccharide head group at *z* = 17 Å from the air/chain interface. On the other hand, Ca^2+^ confined at *z* = 19 Å crosslink charged saccharides and prevent the incorporation of cationic surfactants. We found that the addition of nonlethal aromatic alcohols facilitate the incorporation of cationic surfactants by the significant roughening of the chain/saccharide interface. Combination of precise localisation of ions and molecular-level structural analysis quantitatively demonstrated the synegtestic interplay of ingredients to achieve a high antibacterial activity.

## Introduction

As stated in a report issued by the World Health Organization in 2014, antimicrobial resistance is a major threat to public health^1–3^. This is due to the overuse of therapeutic agents by over-prescribing antibiotics in the clinical treatment of human patients as well as livestock in the farming industry^4,5^. Although the development of antimicrobial agents is necessary for the sustainable society, it is often overlooked that a proper use of sanitisers already enables us to achieve a sufficient hygiene level as well as to reduce the number of antibiotic treatments^6^. Bacteria, including pathogenic and non-pathogenic species, form colonies, often called biofilms, in households, industry sectors, and hospitals. A recent multicentre study reported patients’ bath basins as potential bacterial reservoirs, which may become a source of transmission of nosocomial infections^7^.

**Figure 1:**
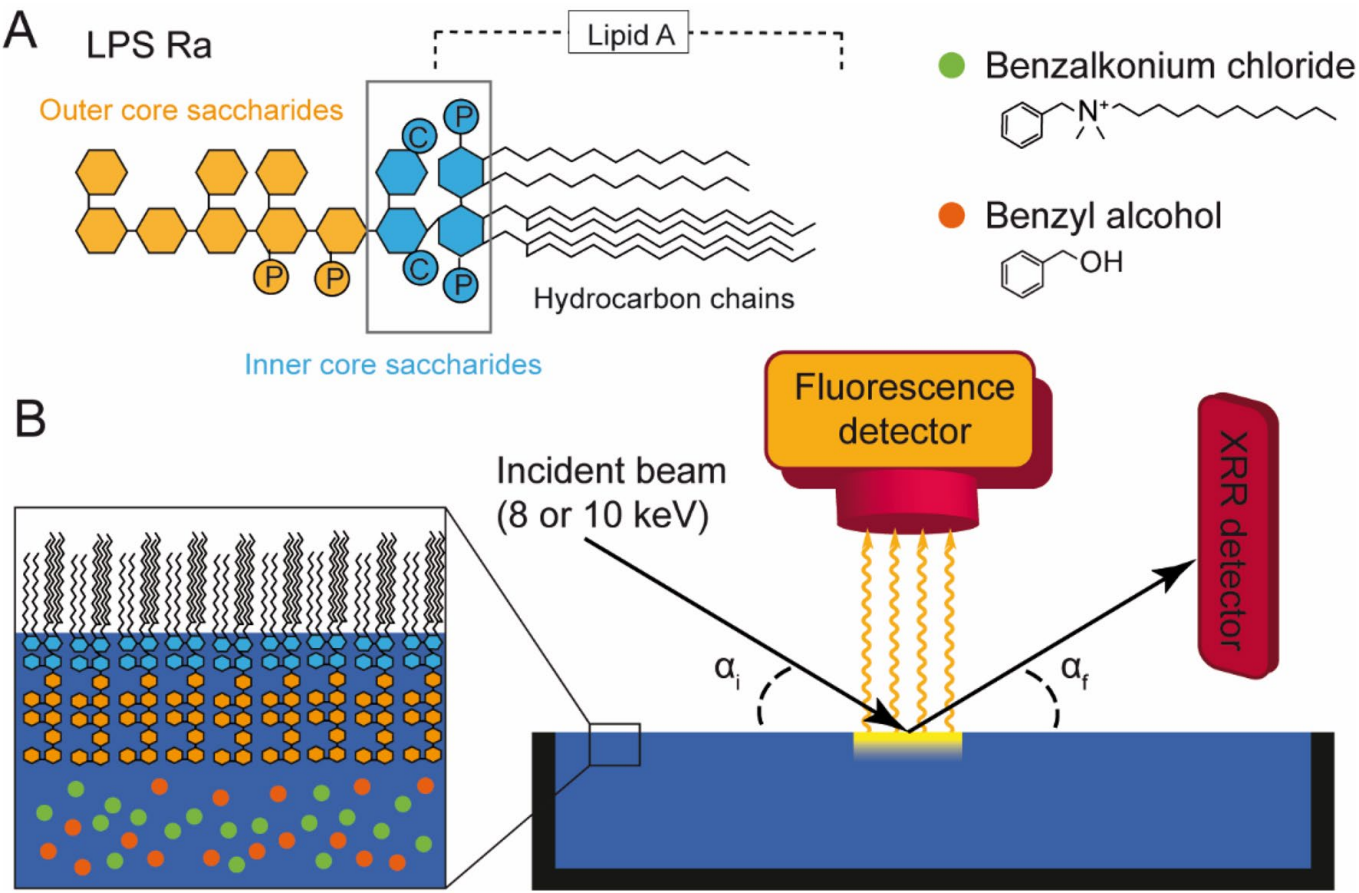
Experimental system and X-ray set up. (**A**) Model of bacterial surfaces based on lipopolysaccharide (LPS Ra). Lipid A is the basic building block, connected to core saccharides. Chemical structures of benzalkonium chloride (BAC) and benzyl alcohol (BzA), two major ingredients of commercial sanitisers. (**B**) Set up for simultaneous X-ray reflectivity (XRR) and grazing incidence X-ray fluorescence (GIXF).

Currently, chemical sanitisers, such as quaternary ammonium compounds (QACs), bisbiguanides (chlorhexidine), and polymeric biguanides, are among the most commonly used disinfectants^6,8^. For example, benzalkonium chloride (BAC, Fig. 1a) is a cationic surfactant widely used as a sanitiser^9,10^. The mechanism of QAC activity is believed to involve (1) the electrostatic binding of positively charged quaternary nitrogen to negatively charged lipids on bacterial membrane surfaces, (2) the integration of hydrocarbon chains of QACs into the hydrophobic core of bacterial membranes, which results in (3) the disruption of cell membranes and subsequent loss of cytoplasm^6,10,11^. However, although QACs have been extensively used since the 1930s, some bacterial strains have gained resistance. For example, *Methylobacterium mesophilicum* KMC10, which form biofilms in bathrooms, are tolerant against BAC treatment up to a concentration of 5 vol%^12^, suggesting that one of the above mentioned steps is hindered. Intriguingly, some alcohols, such as benzyl alcohol (BzA, Fig. 1a), facilitate the antimicrobial function of cationic surfactants against Gram-negative bacteria^11,12^. The combination of cationic surfactants and aromatic alcohols is indeed used in many commercially available sanitisers against biofilms.

Current understanding on the physical mechanism how this combination treatment promotes the antimicrobial function. Yano et al. previously reported that the colony forming unit (CFU) of *Methylobacterium* exposed to either [BAC] = 10 µM or [BzA] = 100 mM alone exhibited no major differences from the control, suggesting that these conditions are nonlethal for bacteria. However, they found that all bacteria died within 3 min of exposure to a BAC and BzA mixture, indicating the synergistic potential of this combination^11^. Using fluorescent probes, they found that the membrane fluidity increased in the presence of nonlethal BzA (100 mM). In line with previous accounts, they interpreted that BzA *destabilised* the membrane structures^13^ and enhanced BAC *permeation*. However, there has been no quantitative study on the influence of BAC and BzA on the stratified membrane structure and ion distribution near the bacterial surface.

Previously, we demonstrated that the monovalent K^+^ ions bound to the saccharide head groups of LPS Ra are displaced by divalent Ca^2+^ ions, resulting in the maintenance of stratified membrane structures even in the presence of cationic antimicrobial peptides, ranging from fish protamines used for food preservation to peptide-based, antiseptic drug candidates^14–17^. Coarse-grained Monte Carlo simulations demonstrated the accumulation of Ca^2+^ ions, which enables the bacterial LPSs to form an electrostatic potential barrier to protect them from the intrusion of cationic antimicrobial peptides^14,18^. Moreover, interfacial shear rheology data indicated that LPS Ra molecules forms a two-dimensional gel in the presence of Ca^2+^ at the air/water interface, while the same membrane behaves as a sol in the absence of Ca^2+^ ions^19^. Based on these data, we hypothesized that the addition of nonlethal aromatic alcohol is necessarry for QAC (BAC) to overcome the barrier made out of negatively charged core saccharides crosslinked by Ca^2+^.

In the present study, we fabricated a model of bacterial outermost surfaces by depositing a monolayer of lipopolysaccharides (LPSs) at the air/water interface, and quantitatively determined how these two compounds influence the structural integrity and specific distribution of each element/ion near the interface. As the most realistic and well-defined building blocks, we chose LPSs purified from *Salmonella enterica* (serovar Minnesota) rough mutant R60 (LPS Ra) As shown in Fig. 1A, LPS Ra has full length of core saccharides like other bioactive wild type LPSs. The LPS Ra molecules possessing uniform saccharide head groups lacking polydispersive O-polysaccharide chains are well suited for XRR structural analysis with slab models^20^. The combination of specular X-ray reflectivity (XRR) and grazing incidence X-ray fluorescence (GIXF)^16,21^ enables us to quantify the detailed structures of LPS membranes in the direction perpendicular to the membrane within sub-Å resolution, as well as the precise position of K^+^ and Ca^2+^ within a Δz = ± 3 Å resolution^17^. First, we assessed bacterial killing by measuring the CFU of *Salmonella enterica* sv. Minnesota Ra in the presence of a commercial sanitiser and mixtures of BAC and BzA. Then, we measured XRR and GIXF of the LPS Ra monolayer in the presence of BAC, BzA, and BAC plus BzA (Fig. 1B). To verify our hypothesis if divalent cations (Ca^2+^) have any protective functions, all experiments were performed in Ca^2+^-free and Ca^2+^-loaded buffers.

## Results

### Antibacterial activity of Bathmagiclean and its ingredients

**Figure 2:**
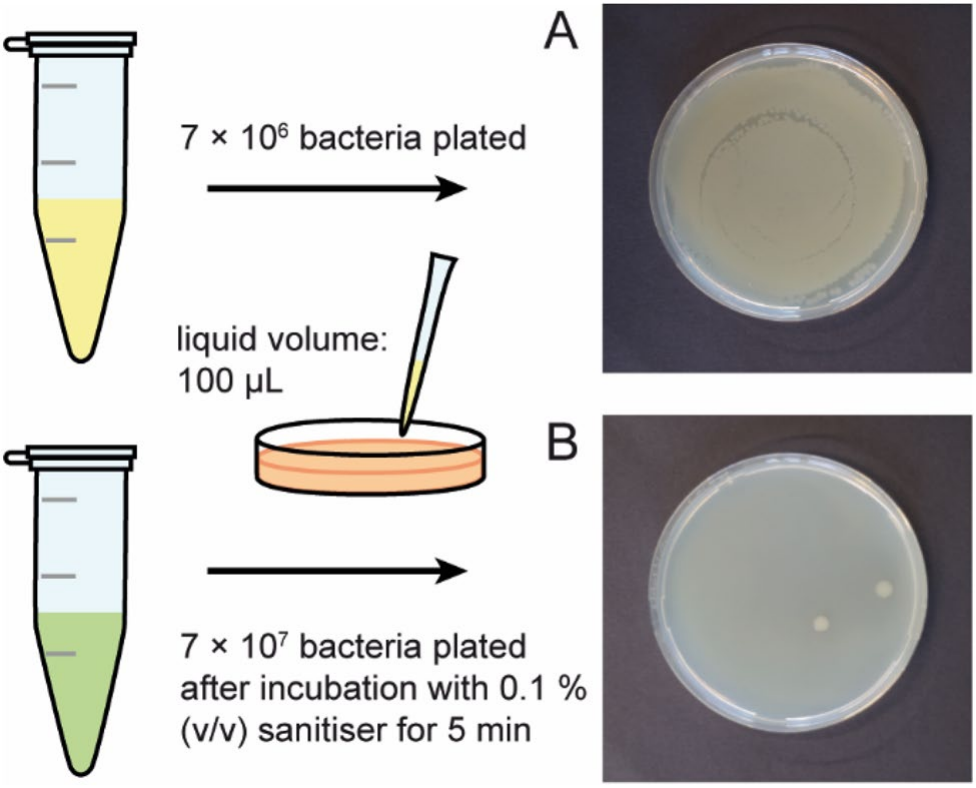
Antibacterial activity of a commercial bathroom cleaner/sanitiser against *Salmonella enterica* (serovar Minnesota) rough mutant R60. (**A**) When the suspension containing 7 × 10^6^ bacteria was deposited on LB agar, the surface was fully covered with the colonies of *Salmonella enterica* after overnight incubation at 37 °C. (**B**) When 10 times more bacteria were exposed to 0.1% (v/v) sanitiser for 5 min before seeding, only 2 bacteria survived after overnight incubation.

First, we performed the positive control experiments using a commercial sanitiser (Bathmagiclean, Kao Corporation, Tokyo, Japan) to confirm its antimicrobial activity to *Salmonella enterica* (serovar Minnesota) rough mutant R60. As shown in Fig. 2A, when a 100-µL portion of suspension containing 7 × 10^6^ bacteria was deposited on Lysogeny broth (LB) agar, the surface was fully covered with bacterial colonies after an overnight incubation at 37 °C. On the other hand, we found that only 2 bacteria survived when 10 times more bacteria were exposed to a 0.1% (v/v) sanitiser for 5 min before seeding (Fig. 2B). The antimicrobial activity of the commercial sanitiser confirmed that the monolayer of LPS Ra is valid as the model to understand how the sanitiser and its ingredients interact with the outer membrane of Gram-negative bacteria.

**Figure 3:**
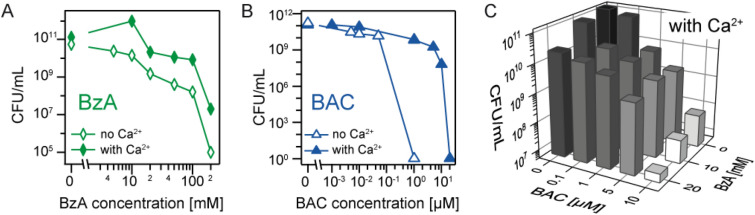
Influence of concentrations on the normalised colony forming unit (CFU/mL) of *Salmonella enterica* (serovar Minnesota) rough mutant. In the presence of (**A**) benzyl alcohol (BzA) and (**B**) benzalkonium chloride (BAC). Buffers: no Ca^2+^ (open symbols) and with Ca^2+^ (solid symbols). Note the difference in the scale of x-axis. (**C**) Synergestic effect of BAC and BzA in killing *Salmonella enterica* in Ca^2+^-loaded buffer.

After confirming the killing capability of the commercial sanitiser containing both BAC and BzA, we measured the normalised colony-forming unit CFU/mL of *Salmonella enterica* after the exposure to solutions of either aromatic alcohol (BzA) or the cationic surfactant (BAC) at different concentrations, in order to understand how each ingredient individually contributes to the killing of bacteria. Figure 3A shows CFU/mL of *Salmonella enterica* after 5 min exposure to BzA solutions of various concentrations in the absence and presence of Ca^2+^ ions. Compared to the control containing intact bacteria CFU/mL_Ca-free_ ≈ 10^12^, the CFU/mL levels decreased but remained high even at high BzA concentrations. For example, CFU/mL_BzA(Ca-free)_ ≈ 10^8^ and CFU/mL_BzA(Ca-loaded)_ ≈ 10^10^ at [BzA] = 100 mM, suggesting that the aromatic alcohol (BzA) alone does not promote the killing of bacteria. Moreover, the CFU/mL levels in Ca^2+^-loaded buffer (solid symbols) were higher than those in Ca^2+^-free buffer (open symbols) at all BzA concentrations, indicating the protective function of Ca^2+^ ions against BzA. All bacteria in Ca^2+^-free buffer died at [BzA] = 200 mM, while those in Ca^2+^-loaded buffer still survived, CFU/mL_BzA(Ca-loaded)_ ≈ 10^7^. Figure 3B represents CFU/mL of *Salmonella enterica* after a 5 min exposure to solutions with different BAC concentrations in the absence and presence of Ca^2+^ ions. We found that all bacteria were already killed at [BAC] = 1 µM in Ca^2+^-free buffer (open symbols), while *Salmonella enterica* could withstand BzA even at 10^5^ times higher dose in the same buffer (Fig. 3A). Conversely, in Ca^2+^-loaded buffer (solid symbols), the CFU/mL still remained high, CFU/mL_BAC(Ca-loaded)_ ≈ 10^10^. In the presence of Ca^2+^ ions, the BAC concentration necessary for killing all bacteria ([BAC] = 20 µM) was more than an order of magnitude higher. The obtained results suggest the critical role of cationic surfactant (BAC) in bacteria killing and the protective function of Ca^2+^ ions.

The next question we wanted to address is if it is necessary to have both BzA and BAC in sanitiser, considering the high efficacy of BAC even in the absence of BzA. We examined if the combination of BAC and BzA has any synergistic effect in Ca^2+^-loaded buffer (Fig. 3C), and found that the CFU/mL level exhibited a clear decrease by adding BzA. The complete killing of *Salmonella enterica* was achieved at [BAC] = 10 µM and [BzA] = 20 mM. As these values are distinctly lower than the individual concentrations of BAC or BzA necessary to kill all bacteria, [BAC] = 20 µM and [BzA] = 200 mM, respectively, our data confirmed that the combination of cationic surfactant and aromatic alcohol indeed facilitates bacteria killing. The same trend was also observed in Ca^2+^-free buffer (Supporting Information Figure S1).

### Influence of aromatic alcohol (BzA) on bacterial outer membrane structures

**Figure 4:**
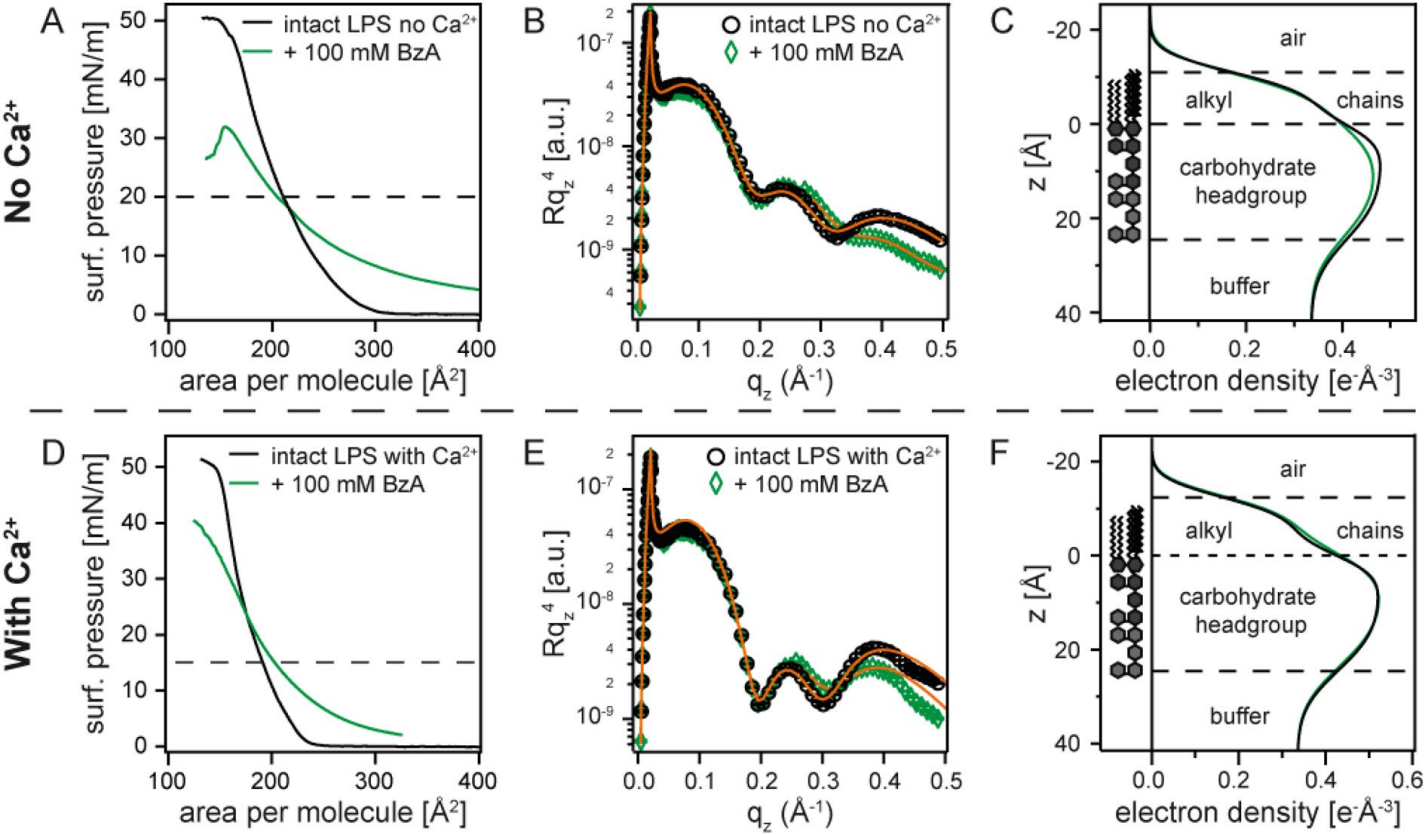
Influence of 100 mM BzA on LPS Ra monolayers. (**A**) Pressure-area isotherms, (**B**) XRR data, and (**C**) reconstructed electron density profiles of LPS Ra monolayers in the absence (black) and presence (green) of BzA measured on Ca^2+^-free subphase. The corresponding data measured on Ca^2+^-loaded subphase are presented in panels (**D**)–(**F**).

To understand the physical mechanism underlying the killing of bacteria, we fabricated a defined model of the outermost surface of bacteria by the deposition of a monolayer of LPS Ra from *Salmonella enterica* (serovar Minnesota) rough mutant at the air/water interface (Fig. 1). Firstly, we examined the influence of 100 mM BzA on the structures of LPS Ra monolayers. To date, many studies have suggested that organic solvents accumulate in bacterial membranes and cause the disorder resulting in increased fluidity^22,23^. Figure 4A shows the pressure-area isotherms of LPS Ra monolayers in the absence (black) and the presence (green) of BzA measured on Ca^2+^-free subphase. XRR measurements were performed at *π* = 20 mN/m, corresponding to the mean molecular area of *A* ≈ 205 Å^2^ (indicated by a broken line). Note that *π* = 0 mN/m of the green curve was defined as the surface pressure of the subphase containing 100 mM BzA (*π*_BzA_ ≈ 15 mN/m). The data including this offset level are presented in Supporting Information Figure S2, confirming that the area per molecule near the collapse converges with that of the intact LPS Ra monolayer. This suggests that LPS Ra molecules dominate the apparent area per molecule at a high compression because BzA molecules near the interface were squeezed out into the subphase. Figure 4B represents the XRR data (symbols) and the corresponding best fit curves (solid lines). The electron density profiles reconstructed from the fitting results are shown in Fig. 4C. As summarised in Table 1, the roughness of the interface between hydrocarbon chains and carbohydrate head groups was slightly elevated from *σ* = 3.9 Å to 5.6 Å by the presence of BzA, suggesting that BzA molecules adsorb to the interface between hydrocarbon chains and carbohydrate head groups and roughen this interface. However, the roughness of the other interfaces showed no remarkable changes. This seems to explain our data showing that 100 mM BzA caused a decrease in CFU/mL in Ca^2+^-free buffer but is not sufficient to kill all bacteria (Fig. 3A).

**Table 1:** The structural parameters of LPS Ra monolayer on Ca^2+^-free buffer, calculated from the best fits of XRR curves.

	*d* [Å]	*ρ* [é Å^-3^]	*σ* [Å]
LPS Ra on Ca^2+^-free buffer			
Alkyl chains	10.6	0.326	3.4
Head group	24.0	0.483	3.9
Subphase	∞	0.334	6.8
LPS Ra on Ca^2+^-free buffer + 100 mM BzA			
Alkyl chains	10.8	0.323	3.9
Head group	24.3	0.472	5.6
Subphase	∞	0.334	6.5
LPS Ra on Ca^2+^-free buffer + 5 µM BAC			
Alkyl chains	11.9	0.377	5.0
Head group	21.6	0.356	7.3
Subphase	∞	0.334	4.3
LPS Ra on Ca^2+^-free buffer + 5 µM BAC + 100 mM BzA			
Alkyl chains	11.7	0.356	4.6
Head group	20.6	0.360	4.9
Subphase	∞	0.334	5.7

The pressure-area isotherms of the LPS Ra monolayer in the presence of Ca^2+^ ions are presented in Fig. 4D. Compared to the corresponding isotherms measured on Ca^2+^-free buffer (Fig. 4A), the influence of BzA seems less on Ca^2+^-loaded buffer. Since the LPS Ra monolayer is compacted in the presence of Ca^2+^ ions^14,17^, XRR measurements were performed at *π* = 15 mN/m, which corresponds to the mean molecular area of *A* ≈ 203 Å^2^ (indicated by a broken line). As presented in Fig. 4E, the difference in the global shape of XRR data caused by the presence of 100 mM BzA was minor in the presence of Ca^2+^. In fact, the electron density profiles reconstructed from the XRR data are almost identical (Fig. 4F). The presence of 100 mM BzA does not cause a roughening of the head–tail interface, *σ* = 3.7 Å, which is different from the results on Ca^2+^-free buffer (Table 2). This finding seems to coincide with the fact that the CFU/mL level in the presence of BzA was about 2 orders of magnitude higher in Ca^2+^-loaded buffer (Fig. 3A), indicating that 100 mM BzA alone is nonlethal for *Salmonella enterica* (serovar Minnesota) rough mutant R60.

**Table 2:** The structural parameters of LPS Ra monolayer on Ca^2+^-loaded buffer, calculated from the best fits of XRR curves.

	*d* [Å]	*ρ* [é Å^-3^]	*σ* [Å]
LPS Ra on Ca^2+^-loaded buffer			
Alkyl chains	12.4	0.319	3.3
Head group	24.6	0.525	3.7
Subphase	∞	0.334	6.7
LPS Ra on Ca^2+^-loaded buffer + 100 mM BzA			
Alkyl chains	12.7	0.348	3.7
Head group	23.0	0.525	3.7
Subphase	∞	0.334	6.8
LPS Ra on Ca^2+^-loaded buffer + 5 µM BAC			
Alkyl chains	12.1	0.292	3.7
Head group	23.7	0.460	4.2
Subphase	∞	0.334	7.2
LPS Ra on Ca^2+^-loaded buffer + 5 µM BAC + 100 mM BzA			
Alkyl chains	10.2	0.313	4.0
Head group	22.9	0.437	6.2
Subphase	∞	0.334	7.9

### Influence of cationic surfactants (BAC) on bacterial outer membranes structures

**Figure 5:**
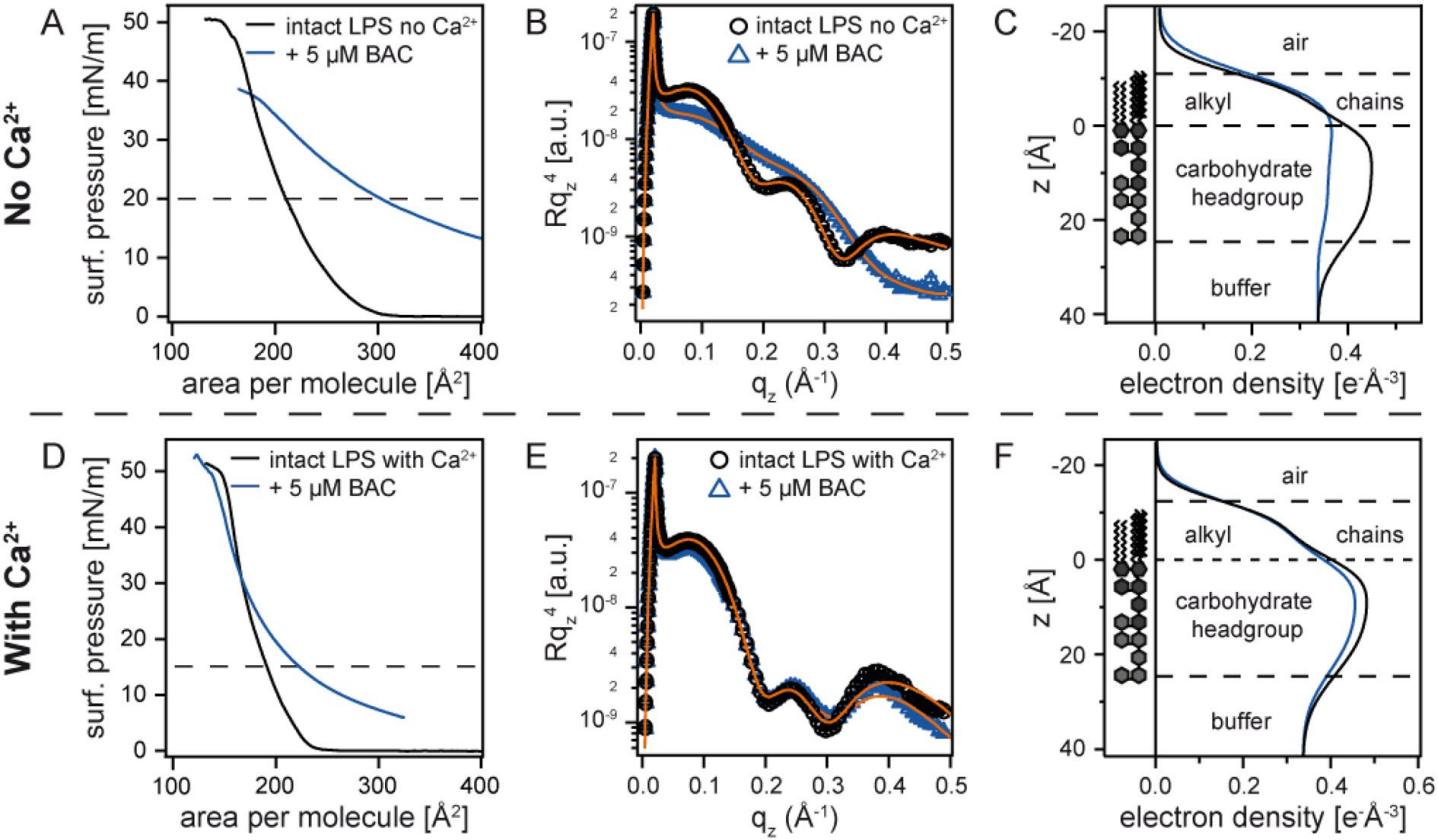
Influence of 5 µM BAC on LPS Ra monolayers. (**A**) Pressure-area isotherms, (**B**) XRR data, and (**C**) reconstructed electron density profiles of LPS Ra monolayers in the absence (black) and presence (blue) of BAC measured on Ca^2+^-free subphase. The corresponding data measured on Ca^2+^-loaded subphase are presented in panels (**D**)–(**F**).

In the next step, we investigated how cationic surfactant (BAC) modulated the layered structure of bacterial membranes with aid of XRR. Figure 5A represents the pressure-area isotherms of LPS Ra monolayers in the absence (black) and presence (blue) of 5 µM BAC measured on Ca^2+^-free buffer. The surface pressure in the presence of BAC was also corrected to the pressure with respect to the subphase containing BAC (*π*_BAC_ ≈ 7 mN/m). The data including this offset level are presented in Supporting Information Figure S3. Compared to BzA (Fig. 4A), the presence of BAC caused a more pronounced change in the isotherms. For example, the mean molecular area at *π* = 20 mN/m increased by Δ*A* ≈ 90 Å^2^ in the presence of BAC, suggesting the incorporation of the hydrocarbon chains of BAC into the LPS membrane^6^. XRR data in the presence of BAC (Fig. 5B) lost periodic oscillations, which is distinctly different from the intact LPS Ra monolayer. In fact, the reconstructed electron density profile (Fig. 5C) clearly indicates that the structural integrity of the LPS Ra monolayer, the stratified membrane structure with conformal roughness, was significantly disturbed by BAC alone, i.e. with no help of BzA (Table 1). This seems consistent with the fact that no bacteria survived after 5 min incubation only with BAC (Fig. 3B).

Intriguingly, the influence of 5 µM BAC on the isotherms seems less pronounced on Ca^2+^-loaded buffer (Fig. 5D). At *π* = 15 mN/m (indicated by a broken line), the increase in the mean molecular area caused by BAC, Δ*A* ≈ 30 Å^2^, was less than one half compared to the data on Ca^2+^-free subphase (Fig. 5A). The global XRR curve in the presence of BAC resembles to that of intact LPS Ra (Fig. 5E), but the main difference can be seen in the head group region. BAC led to a decrease in the electron density of the head group by ∆*ρ* = 0.065 é Å^−3^ (Table 2), which suggests the decrease in the packing density of negatively charged saccharide head groups by the binding of cationic BAC. On the other hand, we observed no distinct sign that BAC are incorporated into the hydrocarbon chains of LPS Ra. The maintenance of stratified membrane structures with conformal roughness was further confirmed by the reconstructed electron density profiles (Fig. 5F). The higher stability of the LPS Ra monolayer observed on Ca^2+^-loaded subphase seems reasonable from the high CFU/mL level (~ 10^9^) at [BAC] = 5 µM. The fact that 5 µM BAC alone is not able to disturb the stratified LPS Ra membrane structure in the presence of Ca^2+^ coincides with the resistance of bacteria against QAC.

### Synergestic effects of aromatic alcohol (BzA) and cationic surfactant (BAC)

**Figure 6:**
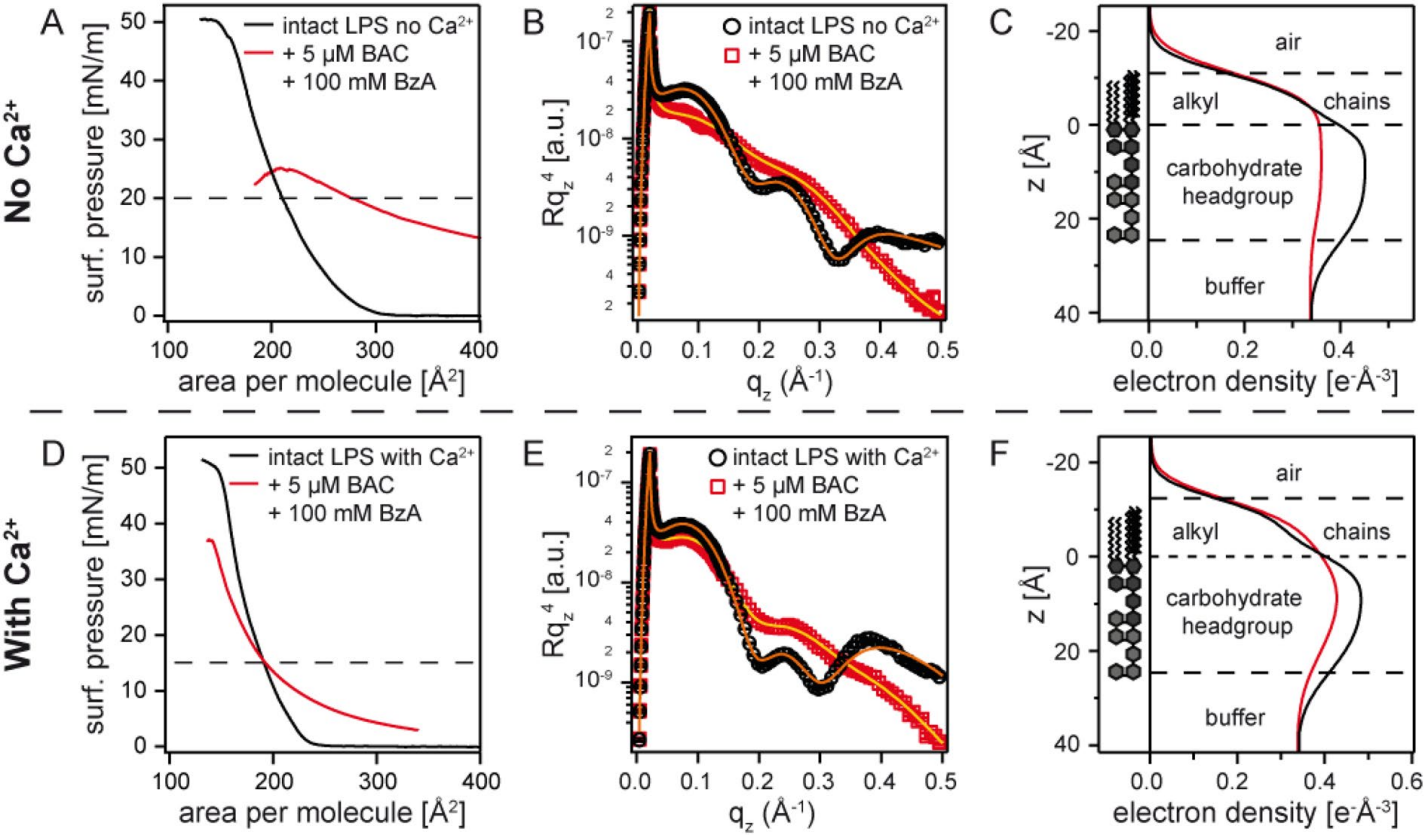
Influence of 100 mM BzA and 5 µM BAC on LPS Ra monolayers. (**A**) Pressure-area isotherms, (**B**) XRR data, and (**C**) reconstructed electron density profiles of LPS Ra monolayers in the absence (black) and presence (red) of BAC and BzA measured on Ca^2+^-free subphase. The corresponding data measured on Ca^2+^-loaded subphase are presented in panels (**D**)–(**F**).

The next question we wanted to address is whether the combination of aromatic alcohol (BzA) and cationic surfactant (BAC) promotes the disturbance of bacterial outer membranes. Figure 6A represents the pressure-area isotherms of LPS Ra monolayers in the absence (black) and presence (red) of 100 mM BzA and 5 µM BAC measured on Ca^2+^-free buffer, which coincides with the composition in a sanitiser. Note that the surface pressure was corrected by that of the subphase containing 100 mM BzA and 5 µM BAC (*π*_BzA+BAC_ ≈ 20 mN/m). The data including the offset level are presented in Supporting Information Figure S4. The addition of BzA to BAC exhibited a more pronounced influence on the isotherm, compared to BAC alone (Fig. 5A). The monolayer exposed to both BAC and BzA was close to collapse at *π* = 20 mN/m, denoting that the LPS membrane was significantly disturbed (Table 1). XRR data collected at *π* = 20 mN/m (Fig. 6B) is similar to that presented in Fig. 5B, implying the loss of stratified membrane structures. This finding seems reasonable, because we observed no bacterial survival in Ca^2+^-free buffer at [BAC] ≥ 1 µM, independent of [BzA] (Supporting Information Figure S1).

Figure 6D shows the corresponding isotherms measured on Ca^2+^-loaded buffer. Remarkably, the global shape of the XRR curve (Fig. 6E) is clearly different from the curves in the presence of only BzA (Fig. 4E) or BAC (Fig. 5E). Most remarkably, we found that the addition of BzA significantly increased the roughness at hydrocarbon chain/head group interface from *σ* = 4.2 Å to 6.2 Å (Table 2). The obtained results therefore clearly indicate the synergestic function of BzA and BAC, where the disturbance of the stratified membrane structures by BzA enables the incorporation of BAC (Table 2).

### Modulation of ion concentration profiles near the interface

**Figure 7:**
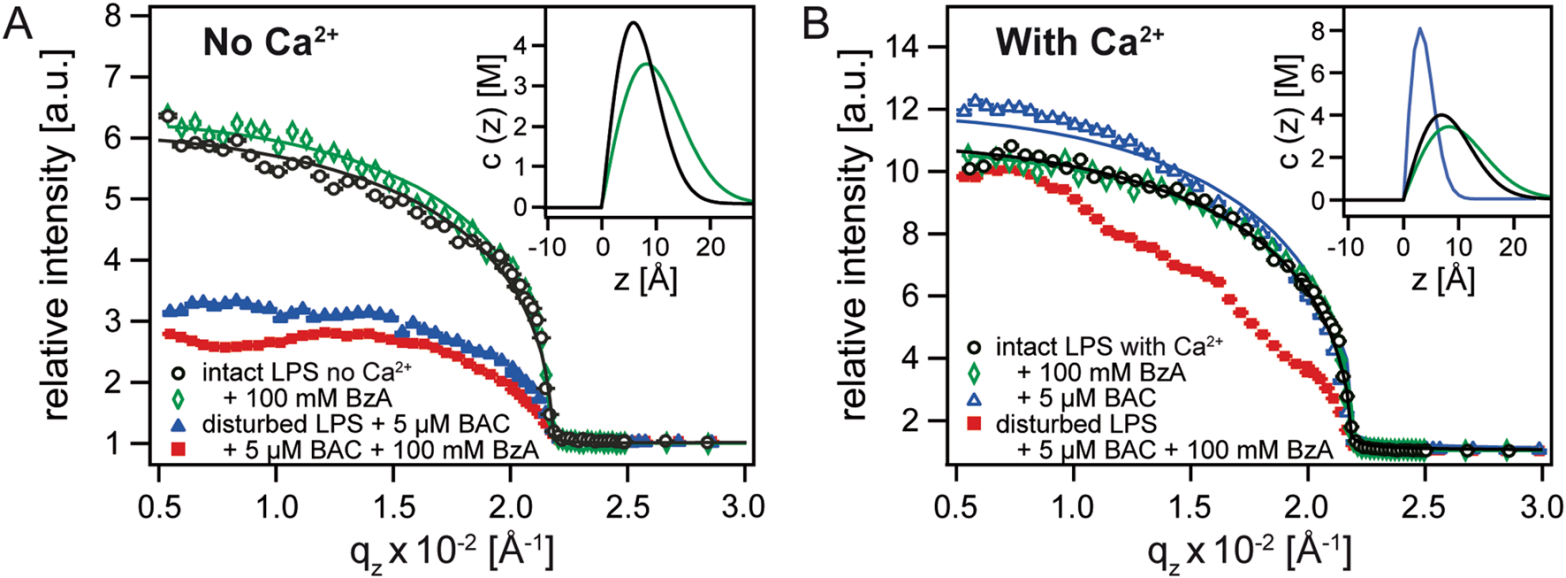
Normalised X-ray fluorescence signals collected at incident angles below and above the critical angle (*q*_c_ = 0.022 Å^−1^). (**A**) K Kα signals measured on Ca^2+^-free buffer, and (**B**) Ca Kα signals measured on Ca^2+^-loaded buffer. Solid lines coincide with the best-fit results of Eq. (1). The analysis of the data presented by only symbols could not achieve reasonable fits due to the disturbance of layered structures, suggested by the XRR data. The ion density profiles near the interface calculated with Eq. (2) are presented in insets.

As a LPS Ra molecule contains charged saccharide units (Fig. 1A), the disturbance of structural integrity of membranes should result in the change in ion distribution near the membrane surface^16,17^. To highlight the protective function of divalent Ca^2+^, we compared the density profiles of K^+^ and Ca^2+^ on Ca^2+^-free and Ca^2+^-loaded subphase. Figure 7A shows the normalised X-ray fluorescence signals from the K Kα line measured on Ca^2+^-free buffer, plotted as a function of *q*_z_. The signals collected below the critical angle (*q*_c_ = 0.022 Å^−1^) are from K^+^ near the interface, while the data from the angle beyond the critical angle reflect K^+^ in bulk. The higher counts at *q*_z_ ≤ *q*_c_ suggests the accumulation of K^+^ near the interface. The solid lines are the best-fit results calculated by Eq. (1). The data from intact LPS Ra (black) and the one in the presence of 100 mM BzA (green) are well fitted, and the corresponding density profiles of K^+^ ions calculated by Eq. (2) are presented in the inset. For an intact LPS Ra monolayer, the concentration of K^+^ ions has its maximum value *c*_max(K)_ = 4.6 M at *z*_max(K)_ = 17 Å from the air/chain interface. The maximum concentration is 46 times higher than the bulk level (100 mM), whereas the peak position corresponds to the position of inner core saccharides consisting of two phosphorylated glucosamine units and two 2-keto-3-deoxyoctonoic acid units. The integration of the concentration along z-axis yields the lateral density of K^+^ ions, *c*_L(K)_ = 2.6 × 10^14^ ions/cm^2^. From the area per LPS Ra molecule at π = 20 mN/m, *A* ≈ 210 Å^2^, the number of K^+^ ions associated with one LPS Ra molecule can be determined to be *N* = 5.5. The obtained value seems reasonable from the net charge per core saccharide, 6 e^−14^. As suggested by XRR (Fig. 4B, C), the roughening of the interface between hydrocarbon chains and head groups caused 100 mM BzA slightly broadens the K^+^ peak (green, inset Fig. 7A). Note the apparent shift in the peak position (Δ*z* = 2 Å) is within the spatial resolution, ± 3 Å^18^. In fact, the calculated lateral ion density (*c*_L(K)_ = 2.9 × 10^14^ ions/cm^2^) and the number of associating K^+^ ions (*N* = 5.7) suggest that the charge neutrality and thus the electrostatic barrier were not disturbed by 100 mM BzA. Although a clear decrease in the counts indicates a distinct decrease in K^+^ ions near the interface, we could not achieve reasonable fitting results for the GIXF data measured in the presence of 5 µM BAC and 5 µM BAC and 100 mM BzA (Fig. 7A). The XRR data suggested that the layered membrane structures were strongly disturbed (Figs. 5, 6).

The normalised X-ray fluorescence signals from the Ca Kα line measured on Ca^2+^-loaded buffer are presented in Fig. 7B. The data from the intact monolayer (black) suggested a distinct condensation of Ca^2+^ near the interface. The concentration profile reconstructed from the GIXF data (inset Fig. 7B) has its maximum almost at the same position, *z*_max(Ca)_ = 19 Å. From the lateral density of Ca^2+^ ions, *c*_L(Ca)_ = 2.5 × 10^14^ ions/cm^2^, we calculated the number of Ca^2+^ ions associated with one LPS Ra molecule, N = 3.9, which is in good agreement with our previous reports indicating that the binding of Ca^2+^ to core saccharides resulted in the displacement of K^+^^16,17^. It is notable that the presence of 100 mM BzA (Fig. 7B, green) caused almost no change, denoting that not only the structures (Fig. 5E, F) but also the ion density profiles near the interface are unchanged in the presence of Ca^2+^. These results suggest that the LPS membrane remained intact even in the presence of 100 mM BzA.

Intriguingly, in the presence of 5 µM BAC, the distribution of Ca^2+^ on Ca^2+^-loaded subphase (Fig. 7B, blue) exhibited a distinct difference from the one of K^+^ measured on Ca^2+^-free subphase (Fig. 7A). The reconstructed ion concentration profile presented in the inset indicates a slight shift in the peak position (Δ*z* = 3 Å), and, more remarkably, the lateral density of Ca^2+^ ions decreased to *c*_L(Ca)_ = 2.4 × 10^14^ ions/cm^2^. As a consequence, the number of Ca^2+^ ions associated with one LPS Ra molecule decreased to N = 3.8, which allows the binding of cationic BAC. When the LPS Ra monolayer is in contact with both 5 µM BAC and 100 mM BzA (Fig. 7B, red), the GIXF analysis could not achieve a reasonable fit due to the loss of distinct layered structures, indicated by the XRR data (Fig. 6E).

## Discussion

Albeit an increasing amount of data has been accumulated on how biophysical characteristics and biochemical functions of bacteria are modulated in response to sanitisers containing cationic surfactants and aromatic alcohols, the molecular-level understanding of how the combination of two ingredients synergistically promotes the killing of bacteria remains uncertain. In this study, we fabricated a defined model of bacteria outer membranes by the deposition of a monolayer of LPSs at the air/water interface. As demonstrated in our previous accounts, the use of LPS molecules purified from wild type strains makes the quantitative structural XRR analysis practically impossible due to long, highly polydispersive O-side chains^24^. To overcome the intrinsic heterogeneity of LPS molecules from wild type bacteria, we used the LPS extracted from a mutant line possessing uniform saccharide head groups, *Salmonella enterica* (serovar Minnesota) rough mutant Ra (LPS Ra). The combination of structure analysis (XRR) and element-specific localisation (GIXF) enabled us to demonstrate how the main ingredients of sanitisers, a cationic surfactant (BAC) and an aromatic alcohol (BzA), modulate the structures and electrostatics of LPS Ra membranes in a quantitative manner. As it has been established that the presence of divalent cations, such as Ca^2+^ and Mg^2+^, facilitates the survival of bacteria, we used Ca^2+^-free and Ca^2+^-loaded buffers to discern the protective role of Ca^2+^. As a control, we performed CFU counting assays (Fig. 3) and verified that the results obtained from the model system can be compared directly with bacterial killing activity.

It is notable that the concentrations of BAC (10 µM) and BzA (100 mM) in sanitisers are not at lethal levels when only one of them is used. Thus, to clearly discriminate the differential roles of the two compounds in the presence and absence of Ca^2+^, we carried out simultaneous XRR and GIXF experiments under a slightly milder condition, 5 µM BAC and 100 mM BzA. Firstly, we examined the solitary effect of 100 mM BzA on the LPS membrane (Fig. 4). The simultaneous XRR and GIXF demonstrated that BzA slightly roughens the interface between hydrocarbon chains and head groups, but the layered structures and the electrostatic barriers remained intact. Our XRR data gave a quantitative answer to the previous report, i.e., the increase in membrane fluidity is caused by the roughening of the interface between the hydrocarbon chains and saccharide head groups. This result is in accordance with previous studies that used NMR^25^ and electrochemical impedance spectroscopy^26^. Compared to BzA, the impact of BAC was more significant (Fig. 5). The isotherm on Ca^2+^-free buffer exhibited a remarkable expansion of the mean molecular area in the presence of 5 µM BAC, and XRR and GIXF suggested that the layered structures of the LPS membrane were significantly disturbed. These results implied the incorporation of hydrocarbon chains of BAC into the LPS monolayer, which is consistent with the fact that no bacteria could survive at [BAC] ≥ 1 µM (Fig. 3B). In contrast, XRR data suggested that BAC alone does not disturb the structural integrity of the LPS Ra membrane on a Ca^2+^-loaded subphase.

Remarkably, our XRR data indicated that the mixture of 100 mM BzA and 5 µM BAC disturbed the stratified LPS membrane structures even on a Ca^2+^-loaded subphase (Fig. 6). This finding provided direct evidence of the synergy of BzA and BAC from a structural viewpoint. This synergestic effect was quantitatively supported by the element-specific localisation of K^+^ and Ca^2+^ using GIXF (Fig. 7). BzA alone did not cause a major change in the concentration profile of ions *c*(*z*) or the number of ions associated with one LPS molecule. On Ca^2+^-free subphase, the presence of BAC alone disabled the calculation of K^+^ density profile, because the layered structure was already disturbed. On Ca^2+^-loaded subphase, the binding of BAC to LPS, suggested by XRR, caused a slight shift in the peak position and a decrease in the number of Ca^2+^ associated with one LPS molecule. However, the “layer” of Ca^2+^ was not disturbed, because the incorporation into the membrane core was blocked by crosslinked core saccharides. In contrast, the ion concentration profiles near the interface could not be calculated in the coexistence of BAC and BzA due to the loss of structural integrity. Interestingly, the concentration of BzA seems to have a significant influence on the synergestic effect; when we exposed the LPS Ra monolayer to 5 µM BAC and 50 mM BzA, we found that the monolayer remained intact in the presence of Ca^2+^ (Supporting Information Figure S5). Thus, we concluded that the roughening of the interface by the binding of BzA to the head/tail interface facilitates the killing of bacteria by the incorporation of BAC into the LPS membrane. These results demonstrated that the structural characterisation by XRR within sub-Å resolution and the element-specific localisation by GIXF within Å resolution is a powerful tool to unravel the physical mechanism of reactions at biological interfaces.

The localisation of various elements within Å resolution opens a large potential to determine the density profiles of not only ions but also molecules. For example, the position and density of proteins bound to the membrane surface can be determined by monitoring S 1S signals from S-containing amino acids^17,27,28^. In May 2020, the National Institute of Technology and (NITE) and the Ministry of Economy, Trade, and Industry (METI) of Japan published the list of surfactants that help the removal of severe acute respiratory syndrome coronavirus 2 (SARS-Cov-2), which includes QAC (https://www.meti.go.jp/english/press/2020/0529_001.html). Simultaneous XRR and GIXF can be used to monitor the initial phase of interactions between virus particles and host cell membranes. Since RNA in coronavirus is surrounded by lipid membranes, the simultaneous XRR and GIXF experiments of membranes with the same composition would help us optimize the molecular structure and dose for the effective virus removal and/or inactivation.

## Conclusions

We unraveled the physical mechanism how cationic surfactants (BAC) and aromatic alcohol (BzA), the main ingredients of commercial sanitisers, synergestically overcome the resistance of bacteria against BAC. Specular X-ray reflectivity (XRR) and grazing incidence X-ray fluorescence (GIXF) were combined to gain the molecular-level structures and ion-specific distributions near the bacterial surface simultaneously. As our previous studies suggested that the crosslinking of charged core saccharides by Ca^2+^ protects the bacterial outermembrane, we hypothesized that the addition of nonlethal (100 mM) BzA facilitates either the binding or incorporation of BAC. In the absence of Ca^2+^, all bacteria (*Salmonella enterica* Minnesota rough mutant) died at [BAC] ≥ 1 µM. In fact, the stratified structures of the model of bacterial outermembranes and the layer of K^+^ ions bound to negatively charged core saccharides (*z* = 17 Å) were significantly disturbed by the exposure to the subphase containing only [BAC] = 5 µM, which corresponds to the actual concentration of BAC in sanitisers. On the other hand, BAC alone were not able to disturb the layered structure. A slight shift of the Ca^2+^ peak and a decrease in the number of associated Ca^2+^ in the presence of BAC indicate that BAC bind to LPS but the membrane incorporation was blocked by negatively charged core saccharides crosslinked by Ca^2+^ ions. Remarkably, the addition of nonlethal BzA (100 mM) disturbed the structural integrity of bacterial outermembranes by the roughening of the chain/saccharide interface. Our XRR data indicated the additional BzA caused a significant increase in the interfacial roughness between hydrocarbon chains and saccharide head groups, which facilitates the incorporation of cationic surfactants.

The function of reagents with mutiple ingredients is often explained by the simple summation of the function of each ingredient. Although commonly used reflectivity-based techniques, such as XRR, are powerful tools to detect subtle changes in the thickness, density and roughness on the molecular level, they are not sensitive to the element specific density profiles in the vicinity of interfaces. Our data demonstrated that the simultaneous XRR and GIXF measurements provide more “element-specific” information, which helps us gain insight into physical mechanisms of reactions at complex, biological interfaces, such as binding of virus particles to the host cell membranes and inactivation of virus by surfactants.

## Materials and methods

### Materials

LPS Ra was purified from the bacterial rough strains of *Salmonella enterica* (serovar Minnesota) Ra R60^29^ and lyophilised as previously reported^30,31^. The lyophilised powder of LPS Ra was dissolved in a mixture of liquid phenol, chloroform, and petroleum ether at a volume ratio of 2:5:8. Benzalkonium chloride (BAC, hydrocarbon chain length: *n* = 12) and benzyl alcohol (BzA) were purchased from Sigma-Aldrich (Munich, Germany). Lysogeny broth (LB) and LB agar were purchased from Sigma-Aldrich (Munich, Germany). Both Ca^2+^-free buffer (100 mM KCl, 5 mM Hepes) and Ca^2+^-loaded buffer (50 mM CaCl_2_, 5 mM Hepes) were adjusted to pH 7.4 by titrating with KOH and Ca(OH)_2_, respectively Throughout this study, double deionised water (Millipore, Molsheim) with a specific resistance of *ρ* > 18 MΩ cm was used. Unless stated otherwise, other chemicals were purchased from Sigma-Aldrich (Munich, Germany) and used with no further purification.

### Antimicrobial activity of commercial sanitiser and ingredients

The bacterial killing assay followed a slightly modified version of a previously reported protocol^11^. In brief, 10 µL of *Salmonella enterica* (serovar Minnesota) rough mutant suspension containing 7 × 10^10^ bacteria/mL was incubated with 1 mL of 0.1% (v/v) bathroom cleaner/sanitiser (Bathmagiclean, Kao Corporation, Tokyo, Japan). To highlight the impact of the two main ingredients, a cationic surfactant (BAC) and an aromatic alcohol (BzA), we also mixed the same bacterial suspensions with 1 mL of BzA and BAC individually and in combination, dissolved in Ca^2+^-free/loaded buffers. The mixture was always incubated for 5 min at 20 °C in a rotator (neoLab Roto-Mix, Heidelberg, Germany) to ensure homogenous mixing. The mixture was serially diluted in LB medium, and each fraction was deposited on an LB agar plate. After an overnight incubation at 37 °C, the colony forming units (CFU) were normalised by volume of bacterial suspension (CFU/mL; Figure S1).

### Isotherms, simultaneous XRR and GIXF measurements

The stock solution of LPS Ra was deposited on the subphase of a Langmuir film balance. The details of membrane preparation is explained in Supporting Information Figure S6. After confirming the complete evaporation of the solvent, the pressure-area isotherms were recorded using a KSV Nima film balance (Biolin Scientific, Vaestra Froelunda, Sweden). For XRR and GIXF experiments, the film was compressed to a surface pressure so that the area per LPS-Ra molecule was comparable for both monolayers on Ca^2+^‏-free and Ca^2+^‏-loaded subphases. The Simultaneous specular X-ray reflectivity (XRR) and grazing incidence X-ray fluorescence (GIXF) experiments were carried out at beamline ID10 of the European Synchrotron Radiation Facility (ESRF, Grenoble). The samples were irradiated with a monochromatic synchrotron beam with a photon energy of either 8 keV or 10 keV (*λ*_8 keV_ = 1.55 Å, *λ*_10 keV_ = 1.24 Å). The film balance was kept in a sealed He gas atmosphere to minimise the scattering of the fluorescence emission by air, and the stability of monolayer was confirmed by monitoring the surface pressure throughout the XRR/GIXF measurement, which takes about 40 min (Supprting Information Figure S7). XRR signals were collected with a linear detector (Mythen 1K, Dectris, Switzerland) following our previous studies^27,28^, and the data were analysed using the Parratt formalism^32^ with a genetic minimisation algorithm implemented in the MOTOFIT software package^33^. GIXF measurements were carried out at incident angles α_i_ across the critical angle of total reflection, $\alpha_{c} = 0.154^\circ$ at 8 keV. X-ray fluorescence signals were recorded with an energy sensitive detector (Vortex, SII NanoTechnology, USA). The fluorescence emission intensities from each characteristic line of elements were extracted by fitting the spectra with multiple Gaussian peaks, as reported previously^18,29^. Fluorescence intensity $I^{ill} (z,\alpha )$ from an element *i* at a distance *z* from the air/water interface at an incidence angle *α* can be written as:

1 $$I_{i}^{f} (\alpha ) = S\int\limits_{0}^{\infty } {I^{ill} (z,\alpha )} c_{i} (z)\exp ( - {z \mathord{\left/ {\vphantom {z {L_{i} }}} \right. \kern-\nulldelimiterspace} {L_{i} }})dz.$$

*S* is a proportional constant that is scaled out after the normalisation. The illumination profile $I^{ill} (z,\alpha )$ can be determined by the matrix propagation technique^34^. The electron density of each slab is obtained from the XRR. The exponential term represents the attenuation of the fluorescence emission between position z and the detector, where $L_{i}$ is the attenuation length of water at the characteristic fluorescence line, such as *L*_K-Kα_ = 68.1 µm and *L*_Ca-Kα_ = 93.7 µm. This enables one to calculate $c_{i} (z)$ the lateral concentration of element *i* at a depth *z*:

2 $$c_{i} (z) = c_{0} + c_{\max } \frac{{\sqrt e \,({\text{z}} - {\text{z}}_{HC} )}}{{{\text{z}}_{\max } }}\exp \left( { - \frac{{({\text{z}} - {\text{z}}_{HC} )^{2} }}{{2{\text{z}}_{\max }^{2} }}} \right).$$

*c*_*0*_ is the bulk concentration, z_*HC*_ the position of interface between hydrocarbon chains and carbohydrate head groups. By assuming an asymmetric Gaussian distribution, one can fit the GIXF data with two parameters using Levenberg–Marquardt nonlinear least square optimisation^35^; (1) the maximum concentration *c*_max_, and (2) the peak position of concentration z_max_.

## Supplementary information

Supplementary information.
