# Supplementary material for: Specific localisation of ions in bacterial membranes unravels physical mechanism of effective bacteria killing by sanitiser
Source: Sci Rep. 2020 Jul 23;10:12302. doi: 10.1038/s41598-020-69064-1 (PMC7378190; doi:10.1038/s41598-020-69064-1)
Supplement: Supplementary file 1 — Supplementary information. [file 41598_2020_69064_MOESM1_ESM.pdf]

Supporting Information

Specific Localisation of Ions in Bacterial Membranes Unravels Physical Mechanism of Effective Bacteria Killing by Sanitiser

Judith Thoma[1,+], Wasim Abuillan[1,*,+], Ippei Furikado[2], Taichi Habe[2], Akihisa Yamamoto[3], Simone Gierlich[1], Klaus Brandenburg[4], Thomas Gutsmann[4], Oleg Konovalov[5], Stefan Kaufmann[1], Shigeto Inoue[2,*], and Motomu Tanaka[1,3,*]

[1] Physical Chemistry of Biosystems, Institute of Physical Chemistry, University of Heidelberg, 69120 Heidelberg, Germany

[2] Analytical Science Research Laboratories, Kao Corporation, 1334 Minato, Wakayama, Wakayama Prefecture 640-8580 (Japan)

[3] Center for Integrative Medicine and Physics, Institute for Advanced Study, Kyoto University, 606-8501 Kyoto, Japan

[4] Research Center Borstel, Leibniz Lung Center, 23845 Borstel, Germany

[5] European Synchrotron Radiation Facility (ESRF), 38043 Grenoble, France

[*]corresponding authors: abuillan@uni-heidelberg.de, inoue.shigeto@kao.com, tanaka@uni-heidelberg.de

[+]these authors contributed equally to this work.

[§]present address: Brandenburg Antiinfaktiva GmbH, 23845 Borstel, Germany

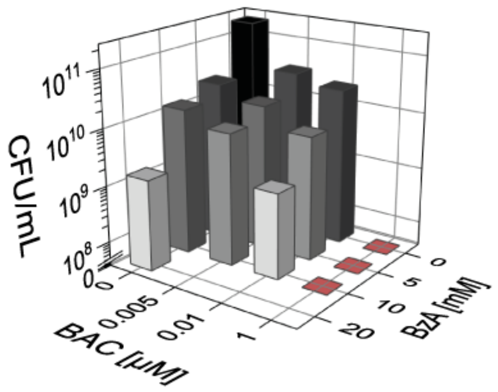

Figure S1. Effect of BAC and BzA on killing of *Salmonella enterica* in Ca^{2+}-free buffer, values of CFU/mL = 0 indicated in red.

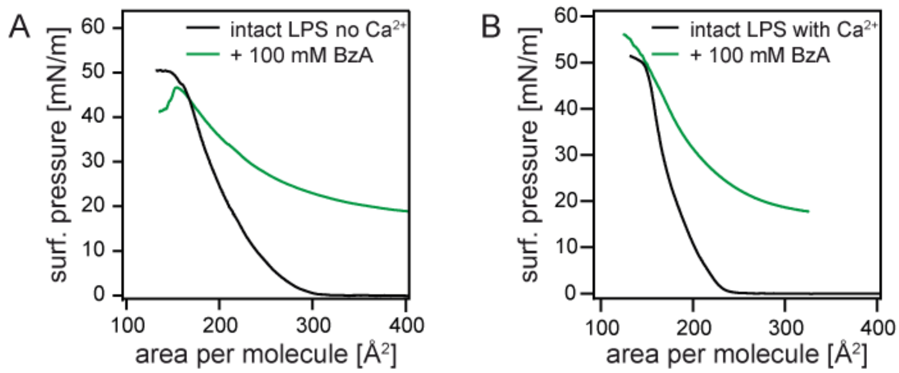

Figure S2. Pressure-area isotherms of LPS Ra monolayers in the absence (black) and the presence (green) of 100 mM BzA measured on (A) Ca^{2+}-free and (B) Ca^{2+}-loaded subphases. The onset caused by BzA ($\pi_{BzA} \approx 15$ mN/m).

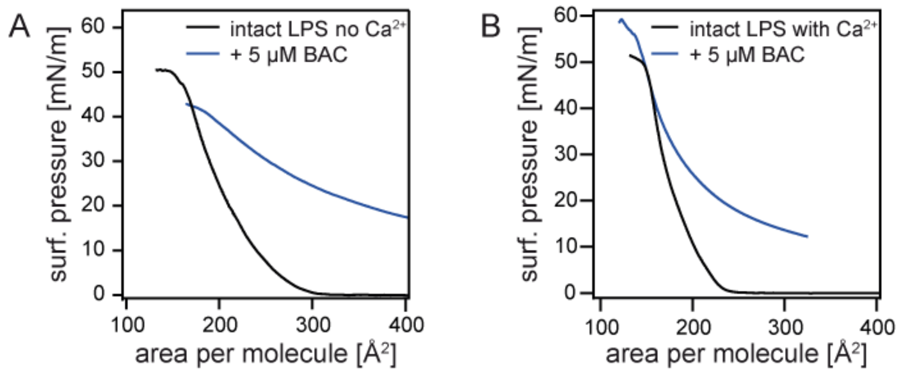

Figure S3. Pressure-area isotherms of LPS Ra monolayers in the absence (black) and the presence (blue) of 5 µM BAC measured on (A) Ca^{2+}-free and (B) Ca^{2+}-loaded subphases. The onset caused by BzA ($\pi_{BAC} \approx 5$ mN/m).

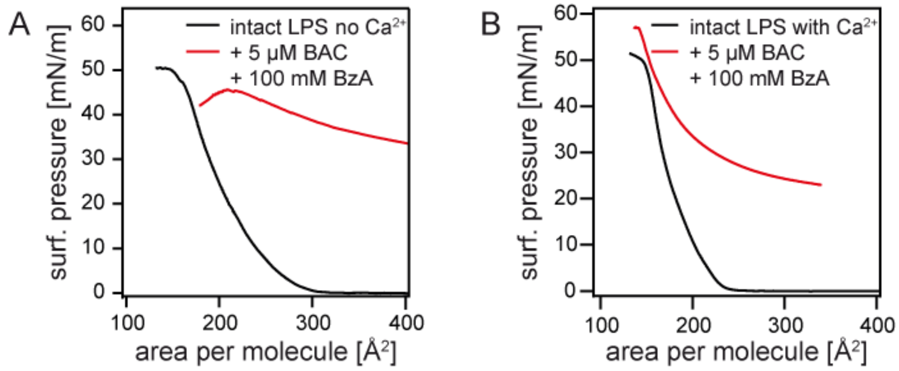

Figure S4. Pressure-area isotherms of LPS Ra monolayers in the absence (black) and the presence (red) of 100 mM BzA and5 µM BAC measured on (A) Ca^{2+}-free and (B) Ca^{2+}-loaded subphases. The onset caused by BzA ($\pi_{BzA+BAC} \approx 20$ mN/m).

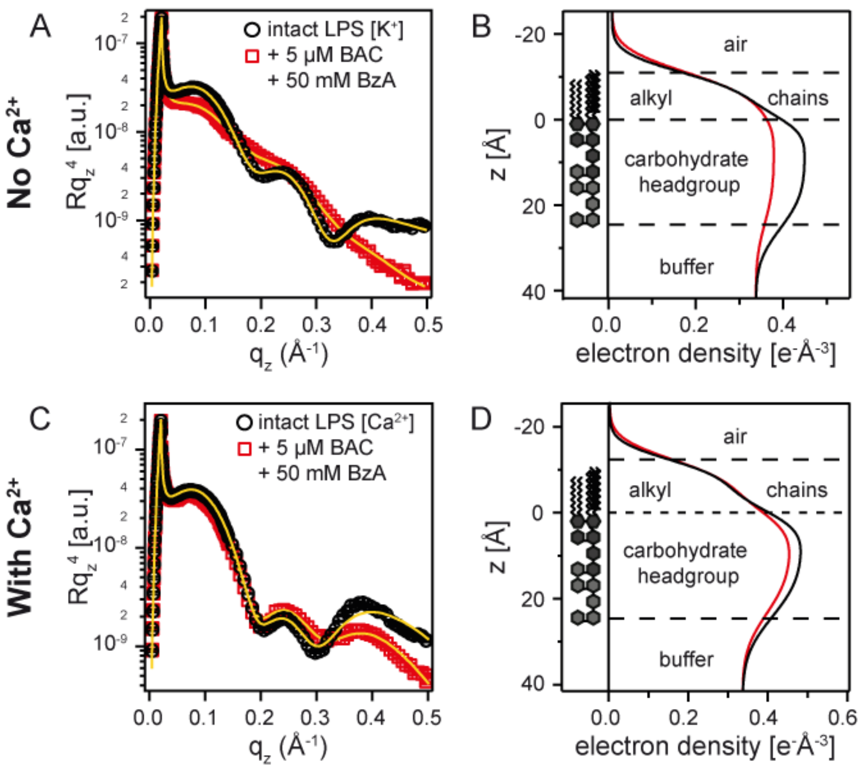

Figure S5. (A) XRR data and (B) reconstructed electron density profiles of LPS Ra monolayers in the absence (black) and the presence (red) of 5 µM BAC and 50 mM BzA measured on Ca^{2+}-free subphase. The corresponding data collected on Ca^{2+}-loaded subphase are presented in panels (C) and (D).

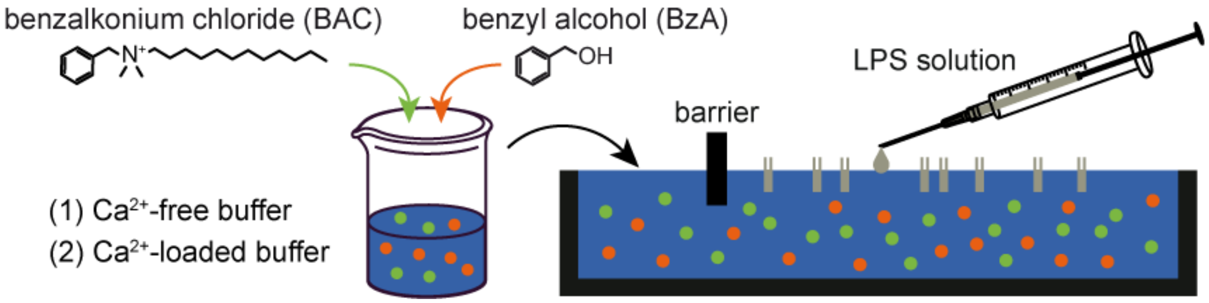

Figure S6: Preparation of Ca^{2+}-free and Ca^{2+}-loaded buffer with and without the addition of benzyl alcohol (BzA) and/or benzalkonium chloride (BAC). Stock solution of LPS Ra, dissolved in a mixture of liquid phenol, chloroform, and petroleum ether at a volume ratio of 2:5:8, was spread on the subphase of the Langmuir film balance containing the previously prepared buffer.

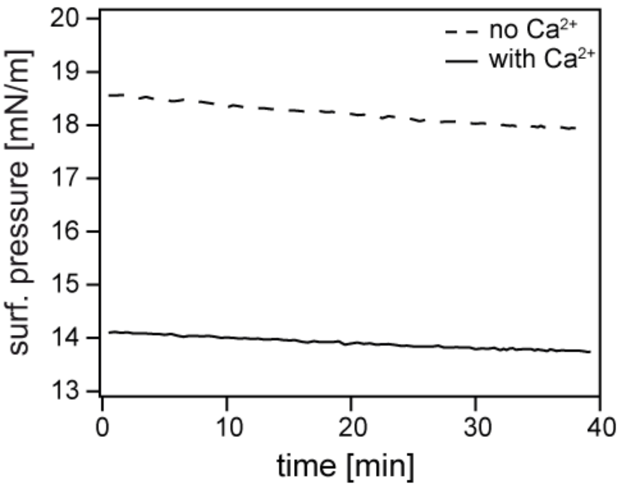

Figure S7: Surface pressure of LPS Ra monolayer on Ca^{2+}-free (broken line) and Ca^{2+}-loaded (solid line) subphase, recorded over 40 minutes. Decrease of surface pressure by 0.6 mN/m in the absence of Ca^{2+}-ions, decrease by 0.3 mN/m in the presence of Ca^{2+}-ions. XRR and GIXF measurements were performed within 10 and 40 minutes, respectively.
